# Soluble Frizzled-7 receptor inhibits Wnt signaling and sensitizes hepatocellular carcinoma cells towards doxorubicin

**DOI:** 10.1186/1476-4598-10-16

**Published:** 2011-02-11

**Authors:** Wei Wei, Mei-Sze Chua, Susan Grepper, Samuel K So

**Affiliations:** 1 Asian Liver Center, Department of Surgery, 1201 Welch Road, Stanford University School of Medicine, Stanford, CA 94305, USA; 2 CellzDirect/Invitrogen, 4301 Emperor Blvd, Durham, NC 27703, USA

## Abstract

**Background:**

There are limited therapeutic options for hepatocellular carcinoma (HCC), the most common liver malignancy worldwide. Recent studies have identified the Frizzled-7 receptor (FZD7), important for activation of Wnt-mediated signaling, as a potential therapeutic target for HCC and other cancers.

**Methods:**

We hypothesized that the extracellular domain of FZD7 (sFZD7) would be a clinically more relevant therapeutic modality than previously studied approaches to target FZD7. We expressed and purified sFZD7 from E. coli, and tested its functional activity to interact with Wnt3, its ability to inhibit Wnt3-mediated signaling, and its potential for combinatorial therapy in HCC.

**Results:**

sFZD7 pulled down Wnt3 from Huh7 cells, and decreased β-catenin/Tcf4 transcriptional activity in HCC cells. *In vitro*, sFZD7 dose-dependently decreased viability of three HCC cell lines (HepG2, Hep40, and Huh7, all with high FZD7 and Wnt3 mRNA), but had little effect on normal hepatocytes from three donors (all with low level FZD7 and Wnt3 mRNA). When combined with doxorubicin, sFZD7 enhanced the growth inhibitory effects of doxorubicin against HCC cells *in vitro*, and against Huh7 xenografts *in vivo*. Reduced expressions of c-Myc, cyclin D1, and survivin were observed *in vitro *and *in vivo*. Additionally, sFZD7 altered the levels of phosphorylated AKT and ERK1/2 induced by doxorubicin treatment *in vitro*, suggesting that several critical pathways are involved in the chemosensitizing effect of sFZD7.

**Conclusions:**

We propose that sFZD7 is a feasible therapeutic agent with specific activity, which can potentially be combined with other chemotherapeutic agents for the improved management of HCC.

## Background

The Wnt/β-catenin signaling pathway is commonly dysregulated in various cancers, including hepatocellular carcinoma (HCC) [1]. Aberrations in this pathway have been established to be critical contributors towards hepatocarcinogenesis [2]. In 18-67% of HCC tumors, activation of this cascade, and subsequent accumulation of nuclear and cellular β-catenin has been observed [3,4]. However, mutations of β-catenin are detected only in 20-30% of HCC [3-7]; and loss-of-function mutation of negative regulators axin1 and axin2 are rare in HCC [8-10]. These observations suggest that other upstream elements may be important in the activation of canonical Wnt/β-catenin during hepatocarcinogenesis, such as promoter methylation of secreted frizzled-related protein (SFRP) members [11], and over-expression of frizzled (FZD) receptors [12-14].

FZDs are frequently upregulated in tumor cell lines and tissues [1]. All ten members of the FZD family have a highly conserved N-terminal extracellular, cysteine-rich domain for Wnt ligand binding, a seven-transmembrane linker domain, and a C-terminal cytoplasmic domain that is essential for receptor signaling [15]. In HCC, FZD7 was shown to be markedly upregulated in four transgenic mouse models of HCC [16], and in human tumors [12,13]. The over-expression of FZD7 in surrounding peritumoral and dysplastic liver tissues suggested its involvement in early events in hepatocarcinogenesis [13,16]. Specifically, functional interaction between FZD7 and the Wnt3 ligand, leading to increased nuclear β-catenin accumulation, has been demonstrated in hepatitis B virus-induced HCC cells [12].

The extracellular domain of FZD receptors serves as binding sites for Wnt ligands (most Wnt ligands will bind to multiple FZDs and *vice versa*). This interaction is indispensable for the activation of Wnt/β-catenin signaling, and interference with this interaction offers a feasible approach to modulate Wnt/β-catenin activation in cancers. SFRPs, encoding only the extracellular domain of FZD, act as natural antagonists of the Wnt/β-catenin pathway by binding to Wnt ligands and inhibiting their interactions with FZDs [17]. Recent studies have shown that the expression of some SFRP proteins are inhibited in HCC tissues due to promoter methylation [14], and that the restoration of SFRP1 could inhibit HCC cell growth by blocking the Wnt/β-catenin pathway [11]. Additionally, artificial transfection of a plasmid expressing the FZD extracellular domain antagonizes canonical Wnt/β-catenin signaling [18] and even induces morphological change and attenuates tumor growth in colon cancer cell lines [19]. More recently, Ueno *et al*. demonstrated that siRNA against FZD7 could decrease survival, invasion, and metastatic capability of colon cancer cells [20].

Our study investigates a more readily translatable method to interfere with FZD7/Wnt3 interaction, by using the extracellular peptide of FZD7 (named soluble FZD7 or sFZD7) expressed and purified from E. coli to inhibit Wnt/β-catenin-mediated signaling in human HCC cell lines. The sFZD7 peptide selectively decreased viability of HCC cells, but not of normal hepatocytes. Additionally, it inhibited downstream β-catenin/Tcf4 interaction and transcriptional activity regardless of β-catenin status (wild-type or mutant). *In vitro *and *in vivo*, sFZD7 enhanced the growth inhibitory effects of doxorubicin, possibly *via *down-regulation of target genes of Wnt/β-catenin signaling (c-Myc, cyclin D1, and survivin), and also *via *modulation of the AKT and ERK pathways. Our data suggest that the sFZD7 peptide may allow improved clinical management of HCC, especially when used in combination with standard chemotherapeutic agents.

## Results

### sFZD7 peptide binds to Wnt3 ligand and suppresses Wnt3-mediated β-catenin transcriptional activation in Huh7 cells

**Figure 1:**
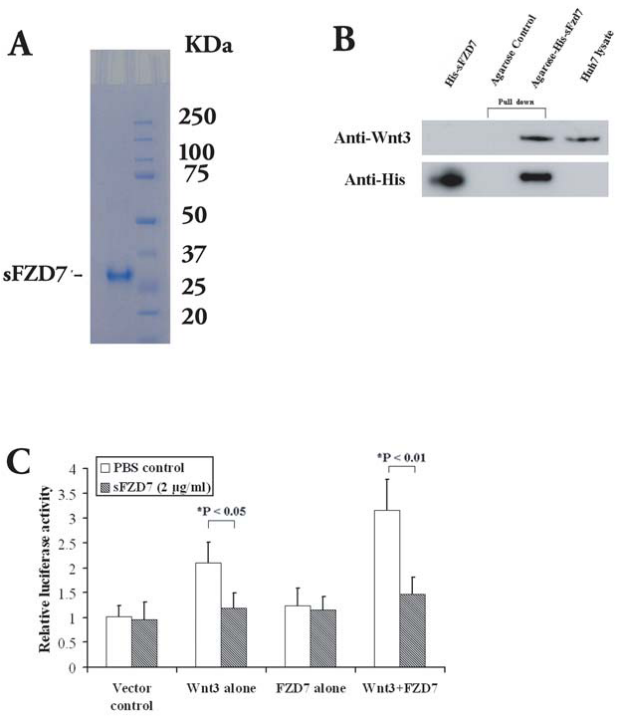
**sFZD7 binds to Wnt3 and suppresses Wnt3-induced β-catenin/Tcf4 transcriptional activation in Huh7 cells**. (**A**). SDS-PAGE analysis of purified sFZD7. (**B**). Pull-down assay demonstrating the interaction between sFZD7 and Wnt3. Huh7 cells lysate was pulled down with Ni-NTA agarose with or without recombinant His-sFZD7 peptide. Protein complexes were centrifuged and eluted, and the supernatants analyzed by Western blot using rabbit anti-Wnt3 antibody (upper panel) and anti-His antibody (lower panel). Huh7 lysate and recombinant His-sFZD7 were used as positive controls. (**C**). sFZD7 abolished the Wnt3-induced β-catenin/Tcf4 transcriptional activity in Huh7 cells. Huh7 cells were transfected with 0.2 μg of each of the indicated expression plasmid, along with 0.3 μg of pTOPFLASH or pFOPFLASH reporter plasmids and 0.1 μg of β-gal to normalize for transfection efficiency. The results are expressed as means ± SD (error bars) of triplicate assays. *P < 0.05 *versus *control.

FZD7 over-expression in HCC implies an activated, FZD7-mediated Wnt/β-catenin signaling [13]. We hypothesized that an extracellular domain peptide of FZD7 (soluble FZD7, sFZD7) will be able to bind extracellular Wnt ligands, thereby reducing ligand interactions with FZD7 and inhibiting Wnt/β-catenin signaling in HCC. Using E. coli, we expressed and purified sFZD7 with an apparent molecular weight of 27 kDa on SDS-PAGE (Figure 1A). As a first step to test the functional activity of sFZD7, we used the pull-down assay to demonstrate that sFZD7 was able to bind to Wnt3 (a reported ligand of FZD7) in Huh7 cells (Figure 1B). Using the β-catenin/Tcf4 transcriptional reporter (TOP/FOPFLASH) luciferase assay in Huh7 cells, we observed that Wnt3 increased β-catenin transcriptional activity, either alone (2-fold, P < 0.05) or in combination with FZD7 (3-fold, P < 0.001). Treatment with sFZD7 significantly abolished the Wnt3-induced activation of β-catenin/Tcf4 transcriptional activity (Figure 1C, *P < 0.05), further confirming that sFZD7 is functionally active.

### sFZD7 inhibits Wnt/β-catenin transcriptional activity and suppresses the expression of downstream oncoproteins

**Figure 2:**
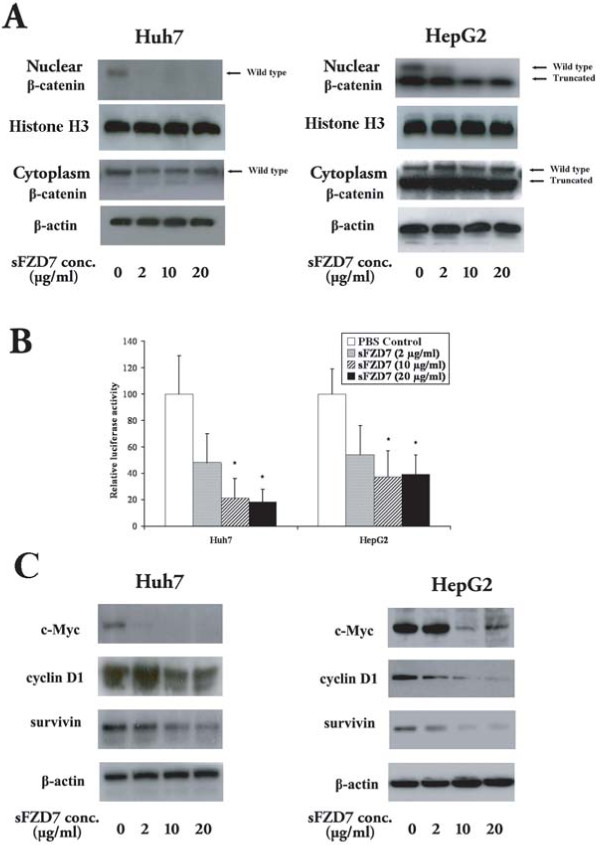
**sFZD7 inhibits Wnt/β-catenin signaling and suppresses the expression of downstream oncoproteins**. (**A**). sFZD7 decreased nuclear β-catenin accumulation but did not decrease cytoplasmic β-catenin in Huh7 and HepG2 cells. Histone-H3 and β-actin were used as loading controls for nuclear and cytoplasmic proteins, respectively. (**B**). Tcf4 reporter assay of Tcf4-dependent transcriptional activity in Huh7 and HepG2 cells. Cells were co-transfected with plasmid encoding β-gal (a control for transfection efficiency) and either the pTOPFLASH or pFOPFLASH reporters. Cells were incubated with control PBS or sFZD7 at various concentrations and harvested after 48 h to measure luciferase and β-gal activities. Reporter gene activation is expressed as relative light units (RLU) detected in pTOPFLASH or pFOPFLASH transfected cells and normalized for β-galactosidase activity. The results are expressed as mean ± SD (error bars). Experiments were performed in triplicates (Independent t-test, *P < 0.05.) (**C**). The effect of sFZD7 on the expression of β-catenin/Tcf4 target genes c-Myc, cyclin D1, and survivin. Huh7 and HepG2 cells were incubated for 48 h with sFZD7 at various concentrations and c-Myc, cyclin D1, survivin, and β-actin (loading control) levels were determined by Western blotting using specific antibodies.

To determine the ability of sFZD7 to disrupt Wnt/β-catenin signaling in HCC cells, we first looked at its effect on nuclear β-catenin accumulation. Treatment of Huh7 and HepG2 cells with different concentrations of sFZD7 for 48 h decreased nuclear β-catenin accumulation in both cell lines, but did not affect cytoplasmic β-catenin level. In HepG2 cells that harbor both wild type and truncated β-catenin [21], sFZD7 decreased the level of wild type β-catenin only, with negligible effect on the truncated β-catenin level (Figure 2A). Additionally, 48 h treatment with sFZD7 caused dose-dependent decreases in β-catenin/Tcf4 transcriptional activities in both Huh7 and HepG2 cells (Figure 2B). Consistently, the endogenous levels of β-catenin/Tcf4 regulated proteins (c-Myc, cyclin D1, and survivin) were reduced after 48 h treatment with sFZD7 (2 μg/ml) in Huh7 and HepG2 cells (Figure 2C). These three proteins are known to be over-expressed in HCC [22-24], and our results indicate that their expression is regulated by Wnt/β-catenin signaling.

### sFZD7 decreases viability of hepatoma cells but not normal hepatocytes

**Figure 3:**
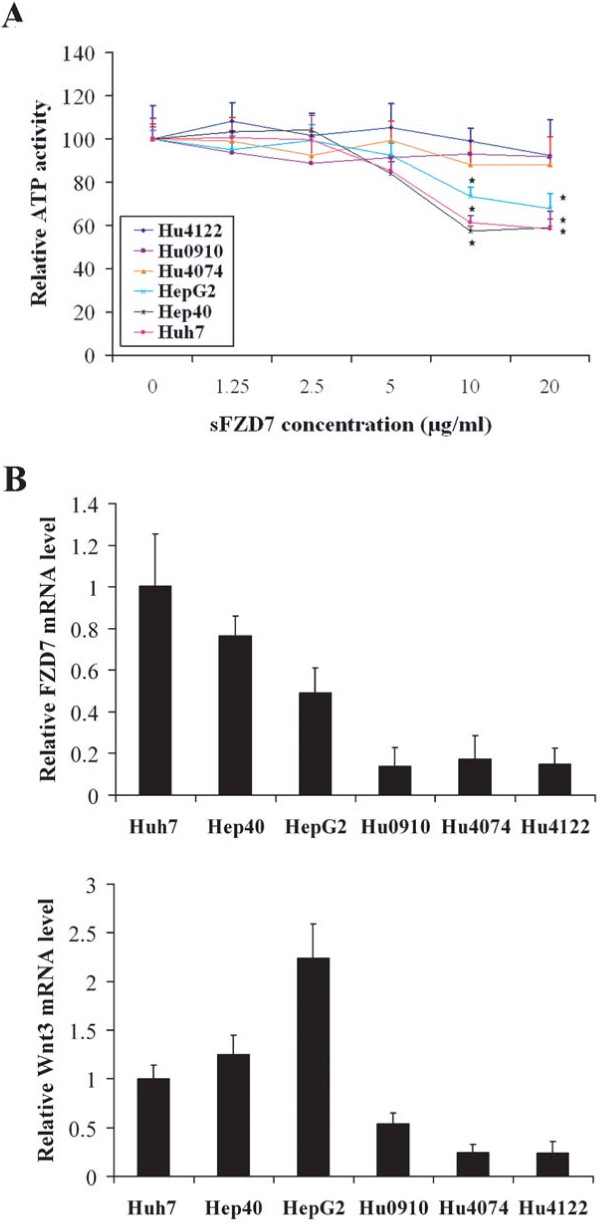
**sFZD7 decreases viability of hepatoma cells but not of normal hepatocytes**. (**A**). Cell viability assay based on cellular ATP content was used to determine the *in vitro *activity of sFZD7 on three human HCC cell lines and normal hepatocytes from three donors following 72 h of sFZD7 treatment (Independent t test, *P < 0.05). Three independent experiments were done, each in triplicates. (**B**). The relative FZD7 and Wnt3 mRNA levels were detected in HCC cell lines and normal hepatocytes from three donors. Data are represented as means ± SD (error bars) from triplicate experiments.

The inhibitory effects of sFZD7 on the growth promoting, Wnt/β-catenin signaling in hepatoma cells suggest that sFZD7 might influence cell viability. We observed that sFZD7 dose-dependently decreased viability of three HCC cell lines (with high FZD7 and Wnt3 expression) after 72 h treatment, but did not affect normal hepatocytes (with low FZD7 and Wnt3 expression) from three donors (Figure 3A). The correlation between the growth inhibitory effect of sFZD7 and cellular FZD7 and Wnt3 mRNA levels (Figure 3B) provide further support that sFZD7 is acting specifically *via *the FZD7-mediated Wnt/β-catenin signaling in HCC cells, and that it exerts preferential activity against HCC cells over-expressing FZD7 and Wnt3.

### sFZD7 sensitizes HCC cells to the anti-proliferative effect of doxorubicin *in vitro*

**Figure 4:**
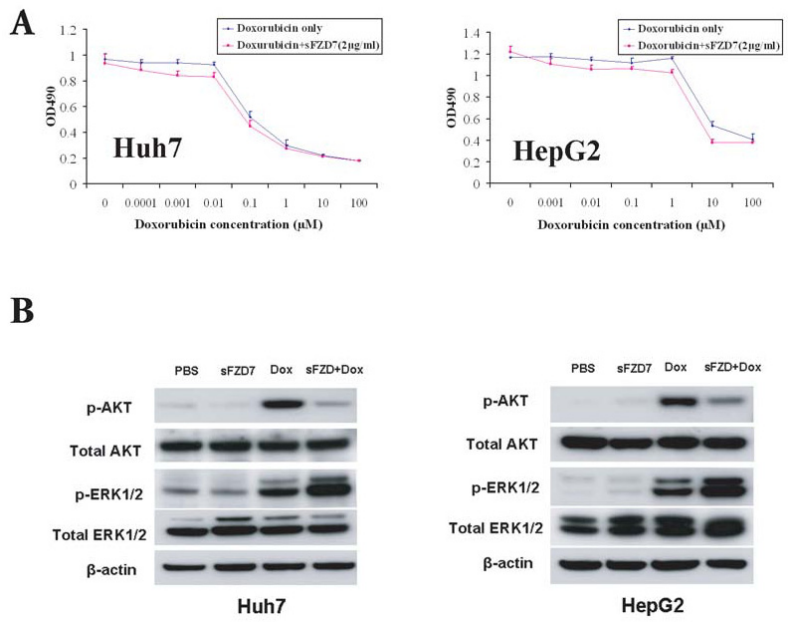
**sFZD7 sensitizes HCC cells to doxorubicin treatment *in vitro***. (**A**). sFZD7 sensitizes HCC cells to anti-proliferative effect of doxorubicin *in vitro*. Huh7 and HepG2 were treated with various concentrations of doxorubicin with or without sFZD7 (2 μg/ml) for 72 h, and cell proliferation determined as described under Materials and Methods. Data are means ± SD (error bars). (**B**). The effect of sFZD7 on the activation of AKT and ERK1/2 induced by doxorubicin. Huh7 and HepG2 cells were incubated with sFZD7 (10 μg/ml) for 48 h, and then doxorubicin (2.5 μM) was added for another 6 h. The phospho-AKT, total AKT, phospho-ERK1/2, total ERK1/2, and β-actin (loading control) levels were determined by Western blotting using specific antibodies.

Since the Wnt/β-catenin signaling pathway has been implicated in chemoresistance of cancer cells [25-28], we next tested whether sFZD7 can sensitize hepatoma cells to the standard chemotherapeutic agent doxorubicin. We demonstrate that sFZD7 (at 2 μg/ml) significantly decreased the IC_50 _value of doxorubicin in Huh7 cells from 0.10 ± 0.02 μM to 0.05 ± 0.01 μM, P < 0.05; and the IC_50 _value of doxorubicin in HepG2 cells from 7.42 ± 0.90 μM to 4.66 ± 0.52 μM, P < 0.05 (Figure 4A, Table 1). Because doxorubicin itself had negligible effects on the expression of c-Myc, cyclin D1 or survivin, we further detected the activation states of AKT and ERK1/2 in Huh7 and HepG2 cells after treatment with sFZD7 and doxorubicin, since these two pathways are also stimulated by Wnt proteins [29]. In both cell lines, doxorubicin alone induced a significant increase in phospho-AKT level (Figure 4B), which was reduced when sFZD7 was combined with doxorubicin (Figure 4B). Total AKT levels were unchanged. Doxorubicin alone also induced the levels of phospho-ERK1/2; however, when sFZD7 was combined with doxorubicin, phospho-ERK1/2 levels were further enhanced without affecting total ERK levels (Figure 4B). Complex cross-talk among these critical pathways (Wnt, AKT, ERK) may together account for the chemosensitizing effect of sFZD7.

**Table 1:** IC_50 _values for doxorubicin with or without sFZD7 in human heptaoma cell lines following 72 h of drug treatment *in vitro*

Cell line	IC_50 _(μM) Doxorubicin only	IC_50 _(μM) Doxorubicin+sFZD7 (2 μg/ml)	Sensitivity Index
Huh7	0.10 ± 0.02	0.05 ± 0.01*	2.00
HepG2	7.42 ± 0.90	4.66 ± 0.50*	1.59

Values are expressed as mean ± SD. The chemosensitizing effects of doxorubicin in combination with sFZD7 are expressed as the sensitivity index (Mean IC_50 _value for doxorubicin alone/Mean IC_50 _value for doxorubicin + sFZD7). Independent T-test was used to compare the IC_50 _values of doxorubicin alone or in combination with sFZD7; * P < 0.05 in both cell lines.

### sFZD7 sensitizes HCC cells to the anti-proliferative effect of doxorubicin *in vivo*

**Figure 5:**
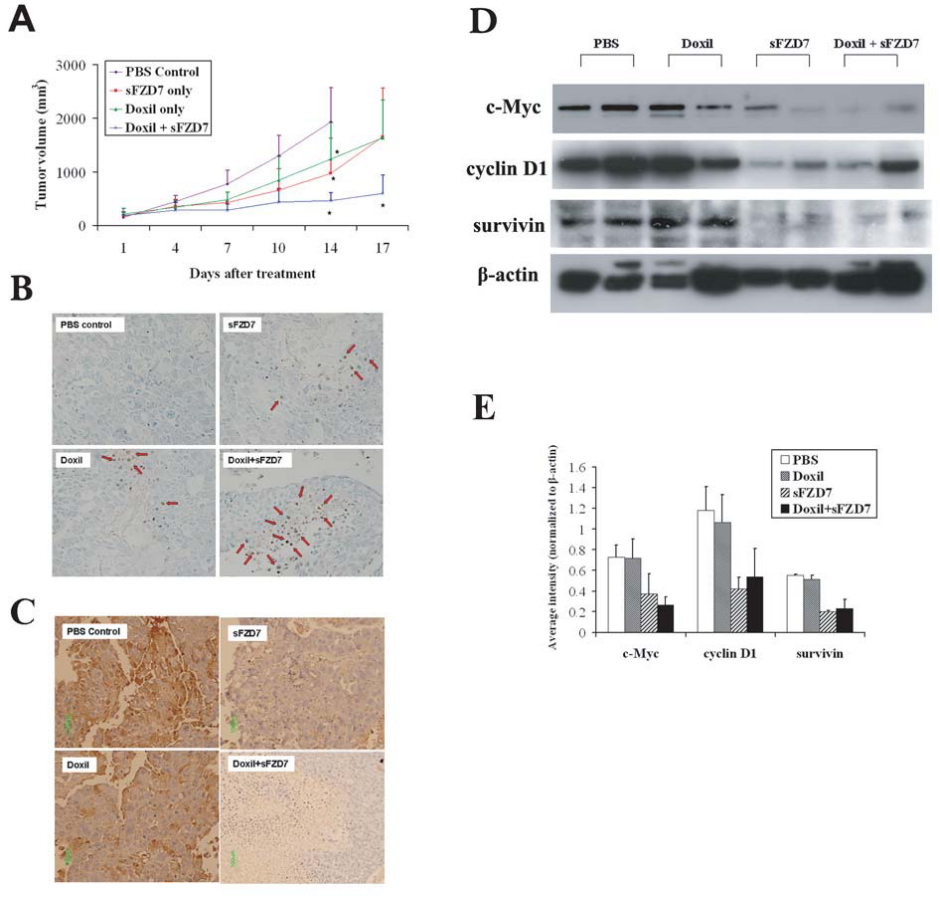
**sFZD7 sensitizes HCC cells to the anti-proliferative effect of doxorubicin *in vivo***. (**A**). Combination of sFZD7 and Doxil enhanced xenograft growth inhibition *in vivo*. Mice bearing Huh7-tumor xenografts were intratumorally injected weekly with PBS control; sFZD7 only (12.5 mg/kg); Doxil only (2.5 mg/kg); or sFZD7 (12.5 mg/kg) combined with Doxil (2.5 mg/kg) (n=5 in each treatment group). Tumor size was measured with digital calipers every three days. Significant differences in the tumor volumes between all treatment groups and the PBS control were observed after 14 days of treatment (*P < 0.05). Additionally, the sFZD7 plus Doxil combination group showed significant differences in tumor volumes compared with sFZD7 only or Doxil only groups after 17 days of treatment (*P < 0.05). (**B**). TUNEL staining of xenograft specimens removed from PBS control and all treatment groups (200 × magnification). Red arrows indicate some positively stained, apoptotic cells. (**C**). Representative cyclin D1 immunostaining of xenograft specimens removed from PBS control and all treatment groups are shown (200 × magnification). (**D**). Protein levels of c-Myc, cyclin D1, survivin, and β-actin (loading control) in tumor xenografts from two mice in each group were determined by Western blotting using specific antibodies. (**E**). The expression levels of c-Myc, cyclin D1, survivin were determined by analyzing Western blots with the ImageJ software, and normalizing their signal intensities to β-actin.

*In vivo*, 14-days treatment with sFZD7 only, Doxil only, or sFZD7 combined with Doxil caused significantly delayed tumor growth in Huh7 xenografts in nude mice, when compared to PBS control (Figure 5A) (independent sample t-test, P = 0.048 for sFZD7 only; P = 0.041 for Doxil only; P = 0.001 for sFZD7 plus Doxil). Additionally, the sFZD7 plus Doxil group showed significantly reduced tumor growth when compared with other treatment groups at day 17 of treatment (P = 0.041, compared with sFZD7 only; P = 0.035, compared with Doxil only).

Tumor xenografts were harvested for TUNEL staining at the end of the treatment period, and apoptotic cells were detected in tumors treated with sFZD7, Doxil, or sFZD7 combined with Doxil (Figure 5B). Whereas the PBS control tumors have less than 10%  apoptotic cells, there were between 10-30% apoptotic cells in the sFZD7 or Doxil single  treated tumors, and more than 50% apoptotic cells in the sFZD7 and Doxil combined  treated tumors. The expression of c-Myc, cyclin D1, and survivin were visualized by immunostaining and Western blotting (Figure 5C-E). While Doxil itself had negligible effect on the expression of c-Myc, cyclin D1, or survivin, sFZD7 alone or in combination with Doxil decreased the tumor levels of these three downstream target proteins of Wnt/β-catenin signaling. This is consistent with our *in vitro *observations that sFZD7 inhibited β-catenin/Tcf4 mediated transcriptional activity, which might contribute to the observed tumor growth inhibition by sFZD7, and its chemosensitizing effect with Doxil.

## Discussion

HCC patients typically have a dismal prognosis, especially if they are diagnosed late. The rising incidence of HCC (the fifth most common cancer globally), and the current lack of effective systemic treatment options, makes it imperative to develop novel and efficacious treatment strategies to overcome current challenges, which include intrinsic resistance to standard chemotherapeutic agents. Recent advances in HCC pathology have identified activated signaling pathways as new therapeutic targets [30-32]. The Wnt/β-catenin pathway and its many components are especially attractive targets because of their functional importance in hepatocarcinogenesis. Among them, FZD7 has been considered a promising therapeutic target [20,33] because FZD7 mediated Wnt/β-catenin signaling is closely associated with growth and survival of tumor cells [8,34]. Additionally, elevated FZD7 mRNA levels have been reported in HCC and other cancers [12,13,35-39]. Our study explores the use of a purified, soluble ectodomain peptide of FZD7 (sFZD7, containing the cysteine-rich domain that interacts with Wnt ligands) as a new therapeutic modality for inhibiting FZD7/Wnt-mediated signaling in HCC.

We first confirmed the functional activity of sFZD7 by demonstrating its ability to bind to its known ligand, Wnt3, using a pull-down assay in Huh7 cells. Additionally, sFZD7 blocked Wnt3-induced β-catenin/Tcf4 transcriptional activation in Huh7 cells, confirming its functional activity. In Huh7 and HepG2 cells, sFZD7 caused dose-dependent inhibitions of nuclear β-catenin accumulation and β-catenin/Tcf4 transcriptional activity, together with reduced expressions of downstream target genes c-Myc, cyclin D1, and survivin. These effects of sFZD7 might underlie its growth inhibitory effects against three hepatoma cell lines (with high expression of FZD7 and Wnt3 mRNA), but not against normal primary hepatocytes (with low expression of FZD7 and Wnt3 mRNA). Our results also suggest that sFZD7 exerts inhibitory effects on Wnt/β-catenin signaling and cell growth regardless of β-catenin status (wild type or mutant). HepG2 cells, which are known to have a constitutively active, truncated mutant of β-catenin lacking the GSK-3β regulatory site [21], respond similarly to sFZD7 treatment as do Huh7 cells with wild type β-catenin. Whether the truncated β-catenin could be regulated by upstream events remains controversial. Shih *et al*. observed that restoration of SFRP attenuated Wnt signaling in Huh6 cells (with a β-catenin point mutation), but not in HepG2 cells with truncated β-catenin [11]. However, our findings are consistent with those of Hocevar *et al*., who observed that the stable over-expression of Dab2, which stabilizes axin as a binding partner of Dvl-3 and axin, decreased nuclear β-catenin accumulation and inhibited β-catenin transcriptional activity in HepG2 cells [40].

In addition to its fundamental roles in tumor cell growth and survival, the Wnt/β-catenin signaling pathway is also implicated in the development of chemoresistance in a wide variety of cancer cells [25-28]. Since HCC is highly chemoresistant and lacks effective therapeutic options [41], we tested the ability of sFZD7 to sensitize HCC cells to treatment with doxorubicin, commonly used for treating HCC. *In vitro*, sFZD7 sensitized Huh7 and HepG2 cells to the anti-proliferative effects of doxorubicin. *In vivo*, combined sFZD7 and Doxil treatment significantly augmented the growth inhibitory effects of either sFZD7 alone or Doxil alone on HCC xenografts. This observation may in part be due to the ability of sFZD7 to reduce the expression of survivin, a Wnt target gene that is over-expressed in HCC, and also a key regulator of cell division and inhibitor of apoptosis that has been demonstrated to be involved in tumor chemoresistance [42]. Additionally, we found that sFZD7 was able to decrease the levels of phospho-AKT induced by doxorubicin alone. This concurs with a recent report by Ohigashi *et al*, who showed that inhibition of Wnt signaling by the Wnt inhibitory factor-1 down-regulated the AKT pathway, leading to enhanced chemosensitivity in PTEN-null prostate cancer cells [43]. Activation of the AKT pathway has been reported to be involved in the activation of Wnt/β-catenin signaling pathway [29] and implicated in the development of acquired drug resistance [44]. Thus, we propose that sFZD7 might decrease survivin expression and inhibit the activation of AKT pathway, thereby contributing towards doxorubicin sensitization in HCC cells.

Additionally, our results allude to complicated cross-talks among critical pathways (Wnt, AKT, ERK) that are involved in HCC. An increase in phosphorylation of ERK1/2 was previously observed in HCC cells treated with doxorubicin, as early as 30 min and up to 24 h after treatment, while total ERK levels were unchanged [45]. Earlier studies in other cell types concur with the ability of the doxorubicin and other DNA-damaging agents (such as etoposide) to induce ERK activation [46-49]. Activation of ERK in response to DNA damage might occur *via *p53-dependent or p53-independent (ATM-dependent) pathways, which then act cooperatively in enhancing cell cycle arrest and apoptosis [48-51]. In our study, sFZD7 further increased the ERK activation induced by doxorubicin in both HepG2 (wild-type p53) and Huh7 (mutant p53) cells, and may enhance sensitivity to doxorubicin by increasing cell cycle arrest and/or apoptosis.

## Conclusion

Given the emerging interest in peptide-based cancer therapeutics, and recent advances in improving peptide stability and delivery [52,53], we propose that the sFZD7 peptide is a feasible therapeutic agent that can be used to specifically abolish the functional activity of FZD7 in HCC. It has potential wide applicability in the treatment of HCC, especially when used in combination with agents targeting other components of the Wnt/β-catenin signaling pathway, or with standard chemotherapeutic agents to increase drug sensitivity by modulating the activities of essential pathways in HCC. Further clinical translation of the sFZD7 peptide will require optimization of the peptide size and physicochemical properties to achieve desirable bioavailability [54].

## Materials and methods

### Cell lines and primary cultures of hepatocytes

Human hepatoma cell lines (Huh7, HepG2, Hep40) were maintained in Dulbecco's Modified Eagle's Medium (DMEM) supplemented with 10% fetal bovine serum (FBS), 100 μg/mL penicillin and 100 μg/mL streptomycin. All media and supplements were from Invitrogen (Carlsbad, CA). Cells were maintained at 37°C in a humidified atmosphere with 5% CO_2_.

Cryopreserved human hepatocytes, collagen-coated 96-well plates, CHRM Thawing Medium, Cell Plating Medium, and Cell Maintenance Medium were received from CellzDirect/Invitrogen (Durham, NC). Characteristics of the hepatocytes and the plating method are as described previously [55].

### Plasmids

Human Wnt3 cDNA clone pUSEamp-Wnt3 (HA-Wnt3) and the empty vector pUSEamp were from Millipore (Billerica, MA). pCMV6-XL5-FZD7 and the empty vector pCMV-XL5 were from OriGene Technologies (Rockville, MD). TCF/Luc reporter constructs, pTOPFLASH (wild type) and pFOPFLASH (mutant), were from B Vogelstein (John Hopkins Oncology Center, Baltimore, MD, USA). The pet28A-sFZD7 was constructed by cloning the PCR-amplified product of the sFZD7 coding region into the BamHI and HindIII sites of vector pet28A (Merck Biosciences, Darmstadt, Germany). sFZD7 fragment was amplified using template containing full length FZD7 plasmid with the following primers: upstream (5'-TTTGGATCCTGCCAGCCCATCTCCAT-3'), and downstream (5'-TTTAAGCTTCACCGGGTGCGGGCGAAGCGCCTCT-3'). The PCR reaction was performed in a GeneAmp PCR system 2700 (Applied Biosystems, Foster City, CA) under the following conditions: heat activation of the polymerase for 5 min at 94°C, followed by 30 cycles of 95°C for 30 sec, 58°C for 30 sec, and 72°C for 45 sec; with a final extension at 72°C for 10 min.

### Expression and purification of sFZD7

Recombinant His-sFZD7 peptide was produced in E. coli (strain BL21, DE3) with IPTG induction, and purified from the insoluble fraction of the bacterial lysate with Ni-NTA (Qiagen, Carpinteria, CA) following the manufacturer's instructions. Elution was done with 8 M urea elution buffer (pH 4.0). Eluted His-sFZD7 fusion protein was dialyzed against phosphate-buffered saline (PBS) with a stepwise increasing pH gradient (pH 4.0 to pH 7.4) at 4°C. The concentration of final His-sFZD7 fusion peptide was determined by BCA protein assay (PIERCE, Rockford, IL) and purification was confirmed by SDS-PAGE and immunoblotting using HRP-labeled anti-His tag antibody (1:1000, Abcam, Cambridge, MA).

### Pull down assay

Huh7 cells (5 × 10^6^) were lysed in RIPA buffer and the crude lysate was clarified by centrifugation at 12,000 × g for 5 min. Recombinant His-sFZD7 peptide (100 μg) was mixed with 15 μl Ni-NTA agarose beads (Qiagen, Carpinteria, CA) in binding buffer (50 mM NaH_2_PO_4_, 300 mM NaCl, 10 mM imidazole, pH 8.0) and incubated for 1 h with gentle rotation, and then washed with wash buffer (50 mM NaH_2_PO_4_, 300 mM NaCl, 40 mM imidazole, pH 8.0). The Ni-NTA agarose with recombinant His-sFZD7 peptide was mixed with Huh7 cell lysate; mock agarose was separately mixed with Huh7 cell lysate. Imidazole was added to the mixtures at a final concentration of 40 mM, and incubated overnight with gentle rotation in 4°C. After centrifugation at 400 × g for 2 min at 4°C, supernatants were discarded and the agarose mixtures were washed with binding buffer three times for 5 min each. The agarose mixtures with the protein complex were then eluted with elution buffer (50 mM NaH_2_PO_4_, 300 mM NaCl, 250 mM imidazole, pH 8.0) and the supernatants were resolved on SDS-PAGE. Western blots were done using the rabbit anti-Wnt3 (1:1000, Abcam, Cambridge, MA), and anti-His tag (1:1000, Abcam, Cambridge, MA) antibodies.

### Luciferase reporter gene assay

Huh7 cells (at 50% confluency) were transfected with 0.2 μg of HA-Wnt3 or empty vector and 0.2 μg of pCMV6-XL5-FZD7 or the empty vector, along with 0.3 μg of pTOPFLASH or pFOPFLASH reporter plasmids and 0.1 μg of β-gal to normalize for transfection efficiency. The amount of DNA in each transfection reaction was kept constant by adding an appropriate amount of the empty vector. Transfection was performed using Lipofectamine 2000 (Invitrogen, Carlsbad, CA) according to the manufacturer's instructions. After 4 h, media containing transfection reagent were replaced with new culture media with or without sFZD7 (2 μg/ml). After 48 h, cells were lysed in 100 μl of lysis buffer, and 20 μl aliquots were assayed using the Promega Luciferase assay system, and the Promega β-gal assay system. Relative light units (RLU) were measured and normalized for transfection efficiency using β-gal activity. Final RLU representing Tcf4 transcriptional activity were calculated by subtracting normalized levels obtained with pFOPFLASH from those obtained with pTOPFLASH.

### Protein extracts and immunoblotting

Huh7 and HepG2 cells were seeded at 50% confluency in 6-well plates and incubated at 37°C overnight. Cells were then treated with PBS, or with purified sFZD7 at desired concentrations (2, 10, or 20 μg/ml) for 48 h. Cell monolayers were washed twice with PBS and then lysed in the RIPA extraction buffer. For nuclear β-catenin immunodetection, nuclear extracts were prepared with a NE-PER Nuclear and Cytoplasmic Extraction Kit (Pierce, Rockford, IL). Equal amounts of protein (20 μg) were resolved by SDS-PAGE and Western blots were performed by using the primary antibodies to c-Myc (1:500, Cat. No. 551101, BD Pharmingen, San Diego, CA), cyclin D1 (1:1000, Cat. No. ab6152, Abcam, Cambridge, MA), survivin (1:1000, Cat. No. NB500-201H, Novas Biologicals, Littleton, CO), β-catenin (1:500; Cat. No. SC-7963, Santa Cruz Biotechnology Inc., Santa Cruz, CA), HRP-Histone H3 (1:10000, Cat. No. Ab21054, Abcam, Cambridge, MA) and HRP-β-actin (1:10000, Cat. No. A3854, Sigma-Aldrich, MO). Secondary antibodies (anti-mouse, Cat. No. SC-2005 and anti-rabbit, Cat. No. SC-2004) conjugated with horseradish peroxidase were from Santa Cruz Biotechnology, Inc. (Santa Cruz, CA).

For sFZD7 and doxorubicin combination treatment, Huh7 and HepG2 cells were treated with PBS, purified sFZD7 alone (10 μg/ml for 48 h), doxorubicin alone (2.5 μM for 6 h), or combination of sFZD7 and doxorubicin (10 μg/ml of sFZD7 for 48 h, followed by addition of 2.5 μM doxorubicin for 6 h). Cell monolayers were then lysed in the RIPA extraction buffer. Equal amounts of total cell protein (20 μg) were resolved by SDS-PAGE and Western blots were performed by using the primary antibodies to ERK1/2 (1:1000, Cat. No. 9102, Cell Signaling Technology Inc., Danvers, MA), p-ERK1/2(1:1000, Cat. No. 4370, Cell Signaling Technology Inc., Danvers, MA), AKT (1:1000, Cat. No. 9272, Cell Signaling Technology Inc., Danvers, MA), p-AKT (1:1000, Cat. No. 4058s, Cell Signaling Technology Inc., Danvers, MA).

### Quantitative real-time PCR assay

Total RNA was extracted from hepatoma cells and hepatocytes by using the RNeasy mini kit (Qiagen, Valencia, CA) following the manufacturer's instructions. Concentration and purity of extracted RNA were determined by optical density measurement at 260 and 280 nm. All real-time PCR reagents were from Applied Biosystems (Foster city, CA). Briefly, first-strand cDNA was generated by random primers using Taqman Reverse Transcriptional Reagent, and real-time PCR was performed by using Taqman Gene Expression Assay (Human FZD7 assay ID: Hs00275833_s1; Human Wnt3 assay ID: Hs00229135_m1) and Universal PCR Master Reagent in a Stratagene Mx3000P Q-PCR system (Stratagene, La Jolla, CA). These reactions were incubated at 95°C for 10 min, followed by 40 cycles at 95°C for 15 sec, and 60°C for 1 min. The expression level of FZD7 was measured in terms of threshold cycle value using Stratagene MxPro software and normalized to the internal control, human 18s rRNA (Part No. 4333760F).

### Cell viability assay

Hepatoma cells were seeded in 96-well plates at 3 × 10^3 ^cells/well, and normal hepatocytes seeded at 3 × 10^4 ^cells/well, and incubated overnight at 37°C prior to addition of sFZD7 at desired final concentrations (range from 1.25-20 μg/ml). Cells were further incubated for 72 h before cell viability was assessed using CellTiter-Glo Luminescent Cell Viability Assay (Promega, Madison, WI) according to the manufacturer's instructions. Luciferase activity was measured on a luminometer (Berthold LB-96V) and values were normalized to the ATP activity and compared with the PBS control value, which was set at 100. Three independent experiments were done, each in triplicates.

### Cell proliferation assay

Hepatoma cells were seeded in 96-well plates at 3 × 10^3 ^cells/well, maintained overnight at 37°C, and incubated with doxorubicin (at various concentrations, ranging from 0-100 μM) with or without sFZD7 (2 μg/ml). After 72 h incubation, cell proliferation was monitored by using CellTiter 96^® ^AQueous One Solution Cell Proliferation Assay (Promega, Madison, WI) according to the manufacturer's instructions. Optical density (OD) at 490 nm was read using a microplate reader (BioTek Instruments, Inc., Winooski, VT). The 50% inhibitory concentrations (IC_50_s) were calculated as an estimate of the anti-proliferative effects of doxorubicin alone or in combination with sFZD7.

### Xenografts in nude mice

Animal experiments were approved by the Administrative Panel on Laboratory Animal Care at Stanford University. Nude mice (ATHYMIC NU/NU; Harlan Sprague-Dawley, Indianapolis, IN) at age 4-6 weeks (body weight of 18 to 25 g) were used. Mice were injected subcutaneously at the dorsal region with 5 × 10^6 ^viable Huh7 cells. After two weeks, when tumors reached approximately 0.4-0.5 cm in diameter, mice were randomized into four groups (n = 5 each) to be intratumorally injected with (1). PBS control (100 μl, once weekly); (2). purified sFZD7 (dose:12.5 mg/kg; volume: 100 μl; once weekly); (3). Doxil (dose: 2.5 mg/kg; volume: 100 μl; once weekly); or (4). sFZD7 (dose: 12.5 mg/kg; volume: 100 μl; once weekly) plus Doxil (dose: 2.5 mg/kg; volume: 100 μl; once weekly). Doxil is a liposomal formulation of doxorubicin for intravenous use. Intratumor administration was chosen to mimic the clinically used treatment method of chemoembolization, which delivers cytotoxic drugs directly to the HCC tumor for greater efficacy and reduced toxicity. Tumor size was measured with digital calipers every three days and was calculated using the formula π/6 × larger diameter × [smaller diameter]^2^. Mice in the PBS group were sacrificed after 14 days, whereas mice in other treatment groups were sacrificed after 17 days of treatment. Tumor tissues were harvested for immunohistochemistry as described below, and for TUNEL staining using the ApopTag Peroxidase in Situ Oligo Ligation Apoptosis Detection Kit (Chemicon International, Temecula, CA) according to the manufacturer's protocol. We assessed the  percentage of cells that stained positively with brown nuclei in five randomly selected  areas of 100 cells per area.  Western blot was used to quantify the protein levels of c-Myc, cyclin D1, or survivin in tumor xenografts of two mice in each group. The antibodies used were: HRP-conjugated anti c-Myc (1:1000, Cat. No. ab62928, Abcam, Cambridge, MA), biotin-conjugated anti-cyclin D1 (1:1000, Cat. No. MS-210-B0, Thermo Scientific, Fremont, CA), HRP-conjugated anti-survivin (1:1000, Cat. No. NB500-201H, Novas Biologicals, Littleton, CO), HRP-conjugated β-actin (1:10000, Cat. No. A3854, Sigma-Aldrich, St. Louis, MO), and secondary antibodies (anti-biotin) conjugated with HRP (1:1000, Cat. No. Ab19221-1, Abcam, Cambridge, MA). Western blots were analyzed by ImageJ software, and signal intensities normalized to β-actin.

### Immunohistochemistry analysis

Immunoperoxidase staining of tumor xenografts was performed on formalin-fixed 4-μm tissue section. Briefly, sections were incubated with monoclonal mouse anti-human c-Myc (1:200, Cat. No. 551101, BD Pharmingen, San Diego, CA), cyclin D1 (1:250, Cat. No. ab6152, Abcam, Cambridge, MA), or survivin (1:500, Cat. No. NB500-201H, Novas Biologicals, Littleton, CO) and then washed with PBS. Subsequent procedures were performed using Dakocytomation Envision System-HRP mouse system (Dako, Carpinteria, CA) according to the manufacturer's protocol.

### Statistical analysis

Statistical significance was determined by the independent-sample T-test using the computer SPSS software (version 10.0; SPSS, Chicago, IL). P values less than 0.05 were considered statistically significant.

## Abbreviations

FZD7: Frizzled-7; HCC: Hepatocellular carcinoma; PI: propidium iodide; Tcf4: T-cell factor 4; TUNEL: Terminal dUTP nick-end labeling

## Competing interests

The authors declare that they have no competing interests.

## Authors' contributions

WW contributed to the major part of experimental work, interpreted the results, performed the statistics and drafted the manuscript. MC conceived the study, participated in its design and data analysis, and contributed with scientific discussion and manuscript preparation. SG contributed the normal hepatocytes and provided training and advice on culturing the hepatocytes. SKS is the principal investigator, responsible for conception of the project, designing the experiments, and approving the final manuscript. All authors read and approved the final manuscript.
